# Psychological Pain and Its Associations with Autistic Traits, Anger Rumination, and Tolerance for Mental Pain Among Medical Students

**DOI:** 10.3390/bs16101824

**Published:** 2026-10-06

**Authors:** Fatma Kartal, Gülşen Teksin, Koray Yarız, Hayriye Mihrimah Öztürk

**Affiliations:** 1 Department of Psychiatry, Faculty of Medicine, Kırıkkale University, Kırıkkale 71450, Türkiye; mihrimahozturk@kku.edu.tr; 2 Department of Psychiatry, Faculty of Medicine, Tekirdağ Namık Kemal University, Tekirdağ 59100, Türkiye; gteksin@nku.edu.tr; 3 Department of Psychiatry, Kırıkkale Yüksek İhtisas Hospital, Kırıkkale 71400, Türkiye; korayyariz@gmail.com

**Keywords:** autistic traits, psychological pain, anger rumination, tolerance for mental pain, medical students

## Abstract

This study aimed to examine the relationships among autistic traits, psychological pain, anger rumination, and tolerance for psychological pain in medical students. This single-center, cross-sectional study included 544 medical students at Kırıkkale University Faculty of Medicine, Türkiye (mean age: 22.40 ± 1.91 years; 55.3% women). Participants completed the Autism Spectrum Quotient (AQ), Orbach and Mikulincer Mental Pain Scale-8 (OMMP-8), Tolerance for Mental Pain Scale-10 (TMPS-10), and Anger Rumination Scale (ARS). Higher autistic traits were associated with greater psychological pain and anger rumination and lower tolerance for psychological pain. Students with AQ scores ≥26 had significantly higher psychological pain and anger rumination and lower tolerance for psychological pain than those with AQ scores <26. In multivariable analysis, autistic traits, anger rumination, and tolerance for psychological pain were independently associated with psychological pain. Cross-sectional indirect-effect analyses showed significant indirect effects through anger rumination (*b* = 0.112, 95% CI [0.066, 0.162]) and tolerance for psychological pain (*b* = 0.185, 95% CI [0.127, 0.243]), while the corresponding direct effects remained significant. These findings highlight the relationships among autistic traits, anger rumination, tolerance for psychological pain, and psychological pain in medical students.

## 1. Introduction

Psychological pain is defined as an intense and distressing emotional state experienced by an individual as a result of experiences such as disappointment, lack of love, shame, and failure (Shneidman, 1998; Meerwijk & Weiss, 2011). Psychological pain has been associated with suicidal behavior, and this relationship may emerge independently of psychiatric diagnoses (Verrocchio et al., 2016).

In this context, the concept of tolerance for psychological pain has received increasing attention in recent years. Tolerance for psychological pain is defined as an individual’s capacity to maintain functioning in the face of intense negative emotions and stressful internal experiences (Simons & Gaher, 2005). Previous research has shown that tolerance for psychological pain is negatively associated with the intensity of psychological pain, suggesting that the two are related but distinct constructs (Becker et al., 2019).

Autism spectrum disorder (ASD) is a neurodevelopmental disorder characterized by difficulties in social communication and social interaction, as well as restricted and repetitive patterns of behavior (American Psychiatric Association, 2013). In recent years, it has been suggested that autistic traits should be conceptualized not only as features observed in individuals with a clinical diagnosis of ASD, but also as dimensional characteristics distributed across the general population at varying levels of severity (Constantino, 2018)**.** In this context, autistic traits encompass milder characteristics, including social and communication difficulties, cognitive rigidity, perseverative thinking tendencies, and unusual patterns of behavior (Carpita et al., 2025). A growing number of studies suggest that even subthreshold autistic traits may be associated with adverse psychological outcomes, including loneliness, depression, self-harm behaviors, and suicidal ideation (Dell’Osso et al., 2019; Richards et al., 2019; Takara & Kondo, 2014; Cremone et al., 2024).

Anger rumination is defined as a mental preoccupation with recurrent and difficult-to-control thoughts about anger experiences, anger-provoking events, and their consequences (Sukhodolsky et al., 2001). Anger rumination, which is considered within the broader framework of repetitive negative thinking, has been reported to prolong feelings of anger following anger-provoking events (Buerke & Capron, 2025).

Previous research has linked autistic traits with greater anger rumination, with perseverative tendencies associated with ASD proposed as one possible explanation for this relationship (Pugliese et al., 2015; Patel et al., 2017). Anger rumination may also be relevant to psychological pain. Uğur and Polat (2021) discussed how repetitive cognitive engagement with previous anger experiences may be associated with negative thoughts and how negative emotions such as anger may develop into psychological pain; they also reported a positive association between anger rumination and psychological pain. Together, these findings provide a rationale for examining anger rumination as a potential indirect pathway in the association between autistic traits and psychological pain.

Previous research has suggested that cognitive inflexibility associated with ASD may make it more difficult to disengage from distressing self-referential thoughts and negative experiences (Burrows et al., 2017). Tolerance for psychological pain, in turn, has been conceptualized as a capacity related to emotion regulation and has been shown to be negatively associated with the intensity of psychological pain (Becker et al., 2019). Although the relationship between autistic traits and tolerance for psychological pain has not, to our knowledge, been directly examined, these findings provide a theoretical rationale for investigating tolerance for psychological pain as a potential indirect pathway in the association between autistic traits and psychological pain.

Medical students are considered a psychologically vulnerable group due to intense academic workload, high performance expectations, a competitive educational environment, and chronic stress (Dyrbye et al., 2006; Bailey et al., 2018; Tsegay et al., 2020). Recent studies have shown that autistic traits are significantly associated with depression, burnout, suicide risk, and lower levels of empathy among medical students (Çelikkol Sadıç, 2024). Therefore, medical students may be considered an important at-risk group for examining the relationships between autistic traits and psychological pain, as well as the cognitive-emotional processes that may play a role in these relationships. However, studies examining the relationships among autistic traits, psychological pain, anger rumination, and tolerance for psychological pain remain limited.

The present study aimed to examine the relationships among autistic traits, psychological pain, anger rumination, and tolerance for psychological pain among medical students, with particular attention to the roles of anger rumination and tolerance for psychological pain in the relationship between autistic traits and psychological pain.

The main hypotheses of the study were as follows:

**H1.**  *Higher levels of autistic traits would be associated with higher levels of psychological pain.*

**H2.**  *Higher levels of autistic traits would be associated with increased anger rumination and lower tolerance for psychological pain.*

**H3.**  *Anger rumination and tolerance for psychological pain would have significant indirect effects on the relationship between autistic traits and psychological pain.*

## 2. Materials and Methods

### 2.1. Study Design and Participants

This study was designed as a single-center, cross-sectional, observational study. The study population consisted of medical students enrolled at a medical faculty in Türkiye. Data were collected between February 2026 and May 2026. Medical students aged 18–30 years who met the eligibility criteria were invited to participate in the study, and those who voluntarily agreed to participate were included. Participation was anonymous and voluntary. The reporting of this cross-sectional study followed the Strengthening the Reporting of Observational Studies in Epidemiology (STROBE) guidelines (see Supplementary Materials).

Because no previous study has directly examined the relationship between autistic traits and psychological pain, the sample size was calculated based on the study by Pugliese et al. (2015), which examined perseverative cognitive-emotional processes and anger rumination associated with autistic traits. Using G*Power 3.1 for correlation analysis, with a two-tailed α of 0.05, power of 0.80, and a small-to-moderate effect size (r = 0.25), the minimum required sample size was determined to be 124. With 544 medical students included in the study, the required sample size was substantially exceeded.

A convenience sampling approach was used, whereby medical students were invited to participate through face-to-face announcements during classes and clinical rotations and through student WhatsApp groups. Because the invitations were disseminated through multiple channels and the exact number of students who received the invitation could not be determined, a response rate could not be calculated. Students who responded to the study invitation were subsequently assessed for eligibility. A total of 571 medical students were assessed for eligibility by a psychiatrist. Of these, 25 participants who reported selected chronic medical conditions specified in the study protocol were excluded. These conditions included rheumatoid arthritis, ankylosing spondylitis, ulcerative colitis, Crohn’s disease, hypertension, diabetes mellitus, epilepsy, multiple sclerosis, and thyroid disorders. In addition, two individuals who did not provide informed consent were excluded. After excluding a total of 27 individuals, the final sample consisted of 544 participants. Current psychiatric disorder, history of psychiatric disorder, psychotropic medication use, and history of suicide attempt were not exclusion criteria and were recorded as clinical characteristics. Given their potential associations with psychological pain, these variables were additionally included as covariates in an adjusted multiple linear regression analysis.

### 2.2. Data Collection Instruments

Data were collected using the Sociodemographic and Clinical Characteristics Data Form, the Orbach and Mikulincer Mental Pain Scale-8 (OMMP-8), the Tolerance for Mental Pain Scale-10 (TMPS-10), the Anger Rumination Scale (ARS), and the Autism Spectrum Quotient (AQ).

1. Sociodemographic and Clinical Characteristics Data Form: This was a semi-structured form prepared by the researchers.
2. Autism Spectrum Quotient (AQ): The AQ is a 50-item self-report scale developed by Baron-Cohen et al. (2001) to assess autistic traits in individuals. The Turkish adaptation and psychometric evaluation of the scale were conducted by Aykan and Kalaycıoğlu (2020). The scale consists of five subdomains: social skills, attention switching, attention to detail, communication, and imagination. Higher scores on the subdomains and the total score indicate higher levels of autistic traits. The total score ranges from 0 to 50, and a score of 32 has been proposed as the clinical cut-off point. However, a score of 26 has also been reported to be an appropriate threshold in terms of sensitivity and specificity for screening autistic traits (Woodbury-Smith et al., 2005). In the present study, the scale was used to assess participants’ levels of autistic traits. The Cronbach’s alpha coefficient for the total score of the scale was calculated as 0.638.
3. Orbach and Mikulincer Mental Pain Scale-8 (OMMP-8): The OMMP-8, developed by Casanova et al. (2021), is an 8-item Likert-type self-report scale that assesses individuals’ levels of psychological pain. The Turkish validity and reliability study of the scale was conducted by Demirkol et al. (2024). The scale consists of three subdomains: experience of irreversibility, emotional flooding, and narcissistic wounds. Higher scores on the scale indicate higher levels of psychological pain. In the present study, Cronbach’s alpha coefficients were 0.767 for the experience of irreversibility subdomain, 0.785 for the emotional flooding subdomain, 0.831 for the narcissistic wounds subdomain, and 0.819 for the total scale score.
4. Tolerance for Mental Pain Scale-10 (TMPS-10): The TMPS-10 is a self-report scale developed by Meerwijk et al. (2019) to assess individuals’ levels of tolerance for mental pain. The Turkish validity and reliability study of the scale was conducted by Demirkol et al. (2019). The scale consists of two subdomains: “Managing the Pain” and “Enduring the Pain.” Higher scores on the scale indicate higher levels of tolerance for mental pain. In the present study, Cronbach’s alpha coefficients were 0.781 for the “Managing the Pain” subdomain, 0.562 for the “Enduring the Pain” subdomain, and 0.830 for the total scale score.
5. Anger Rumination Scale (ARS): The ARS is a 19-item self-report scale developed by Sukhodolsky et al. (2001) to assess individuals’ tendency toward ruminative thinking about anger. The Turkish adaptation of the scale was conducted by Satıcı (2014). The scale consists of four subdomains: Angry Memories, Thoughts of Revenge, Angry Afterthoughts, and Understanding the Reasons. Higher scores on the scale indicate increased anger-related ruminative thinking. In the present study, Cronbach’s alpha coefficients were 0.884 for the Angry Afterthoughts subdomain, 0.851 for the Angry Memories subdomain, 0.691 for the Thoughts of Revenge subdomain, 0.752 for the Understanding the Reasons subdomain, and 0.933 for the total scale score.

### 2.3. Ethical Approval

Ethical approval for the study was obtained from the Non-Interventional Research Ethics Committee of Kırıkkale University (Meeting Date: 14 January 2026; Decision No: 2025.12.42). The study was conducted in accordance with the principles of the Declaration of Helsinki. All participants were informed about the study, and informed consent was obtained from all participants prior to participation.

### 2.4. Statistical Analysis

Statistical analyses were performed using IBM SPSS Statistics for Windows, Version 27.0 (IBM Corp., Armonk, NY, USA). Cross-sectional indirect-effect analyses were conducted using Model 4 of the PROCESS macro developed by Hayes (2017).

Descriptive statistics were calculated for the sociodemographic and clinical characteristics of the participants. Categorical variables were presented as numbers and percentages [*n* (%)], whereas continuous variables were presented as mean ± standard deviation.

The normality of the total and subscale scores was evaluated using the Shapiro–Wilk test together with skewness and kurtosis values. Considering the sample size and distributional characteristics of the variables, parametric statistical methods were used.

Comparisons between groups defined according to the AQ cut-off score (AQ < 26 and AQ ≥ 26) were performed using independent-samples t-tests. Homogeneity of variance was assessed using Levene’s test. Relationships among the study variables were examined using Pearson correlation analysis.

To examine variables associated with OMMP-8 total scores, separate simple linear regression analyses were first performed for ARS total, TMPS-10 total, and AQ total scores. Subsequently, a multiple linear regression analysis was conducted to evaluate their independent associations with OMMP-8 total scores. All independent variables were entered simultaneously into the multiple regression model using the Enter method. In an additional adjusted multiple linear regression analysis, current psychiatric disorder, history of psychiatric disorder, psychotropic medication use, and history of suicide attempt were included as covariates. The main study variables and covariates were entered simultaneously using the Enter method. Prior to regression analyses, the assumptions of linearity, normality of residuals, homoscedasticity, independence of errors, and multicollinearity were evaluated. Linearity and homoscedasticity were assessed by visual inspection of standardized residuals plotted against standardized predicted values, while the normality of residuals was evaluated using histograms and normal P–P plots. Independence of errors was assessed using the Durbin–Watson statistic, and multicollinearity was evaluated using tolerance and variance inflation factor (VIF) values. Cook’s distance was additionally examined to identify potentially influential observations. Unstandardized coefficients (*B*), standard errors (SE), standardized regression coefficients (*β*), 95% confidence intervals, and *p*-values were reported.

For the cross-sectional indirect-effect analyses, the first model examined the indirect effect of AQ total score on OMMP-8 total score through ARS total score, whereas the second model examined the indirect effect through TMPS-10 total score. The significance of indirect effects was assessed using 5000 bootstrap samples and 95% confidence intervals. Unstandardized coefficients (*B*), *p*-values, 95% confidence intervals, and *κ*^2^ values as measures of effect size were reported. Statistical significance was set at *p* < 0.05 for all analyses.

## 3. Results

The sociodemographic and clinical characteristics of the participants are presented in Table 1. The mean age of the 544 participants included in the study was 22.40 ± 1.91 years, and 55.3% were women. Of the participants, 53.5% were enrolled in preclinical years. A current psychiatric disorder was reported by 7.7% of the participants, while 11.6% reported having received a psychiatric diagnosis in the past. Psychotropic medication use was reported by 5.7% of the participants, and 12.1% had a family history of psychiatric illness. A history of suicide attempt was reported by 2.2% of the participants (Table 1).

Descriptive statistics for the participants’ scale scores and group comparisons based on the AQ cut-off score are presented in Table 2. The mean scale scores were 20.61 ± 5.38 for the AQ, 10.86 ± 5.60 for the OMMP-8 total score, 36.26 ± 7.05 for the TMPS-10 total score, and 44.04 ± 10.93 for the ARS total score. Compared with the AQ < 26 group, the AQ ≥ 26 group had significantly higher OMMP-8 total scores (*p* = 0.001), significantly lower TMPS-10 total scores (*p* < 0.001), and significantly higher ARS total scores (*p* = 0.027) (Table 2).

The results of the Pearson correlation analysis conducted to evaluate the relationships between participants’ AQ total scores and OMMP-8, TMPS-10, and ARS total and subdomain scores are presented in Table 3. Positive correlations were found between the AQ total score and the OMMP-8 total score (*r* = 0.276, *p* < 0.001), Factor 1 (*r* = 0.294, *p* < 0.001), Factor 2 (*r* = 0.099, *p* = 0.020), and Factor 3 (*r* = 0.291, *p* < 0.001). Negative correlations were found between the AQ total score and the TMPS-10 total score (*r* = −0.289, *p* < 0.001), EP (*r* = −0.251, *p* < 0.001), and MP (*r* = −0.223, *p* < 0.001) scores. In addition, positive correlations were observed between the AQ total score and the ARS total score (*r* = 0.202, *p* < 0.001), Understanding the Reasons (*r* = 0.194, *p* < 0.001), Angry Memories (*r* = 0.182, *p* < 0.001), Thoughts of Revenge (*r* = 0.172, *p* < 0.001), and Angry Afterthoughts (*r* = 0.163, *p* < 0.001), (Table 3).

Simple linear regression analyses were performed separately for ARS total, TMPS-10 total, and AQ total scores to examine their associations with OMMP-8 total scores (Table 4). In the simple regression analyses, TMPS-10 total score was significantly and negatively associated with OMMP-8 total score (*β* = −0.641), whereas ARS total (*β* = 0.568) and AQ total (*β* = 0.276) scores were significantly and positively associated with OMMP-8 total score (all *p* < 0.001). The proportions of variance explained by the individual models were 41.2% for TMPS-10 total (*R*^2^ = 0.412), 32.3% for ARS total (*R*^2^ = 0.323), and 7.6% for AQ total (*R*^2^ = 0.076) (Table 4).

The multiple linear regression model including ARS total, TMPS-10 total, and AQ total scores was statistically significant (*F* = 190.832, *p* < 0.001). The model explained 51.5% of the variance in OMMP-8 total scores (*R*^2^ = 0.515; adjusted *R*^2^ = 0.512). Based on the standardized regression coefficients, TMPS-10 total score showed the strongest independent association with OMMP-8 total score (*β* = −0.466, *p* < 0.001), followed by ARS total score (*β* = 0.345, *p* < 0.001). AQ total score was also independently associated with OMMP-8 total score (*β* = 0.072, *p* = 0.023) (Table 4).

In an additional analysis adjusting for current psychiatric disorder, history of psychiatric disorder, psychotropic medication use, and history of suicide attempt, the associations of ARS, TMPS-10, and AQ total scores with psychological pain remained significant (Table 4). The adjusted model explained 51.8% of the variance in OMMP-8 total scores (*R*^2^ = 0.518, adjusted *R*^2^ = 0.512, *F* = 82.45, *p* < 0.001) (Table 4).

Visual inspection of the residual histograms and normal P–P plots indicated no substantial deviations from normality in either multiple regression model. The standardized residual-versus-predicted value plots showed no clear systematic patterns suggesting substantial violations of linearity or homoscedasticity. Durbin–Watson statistics were 1.867 for the primary model and 1.874 for the adjusted model. Tolerance values ranged from 0.757 to 0.910 and VIF values from 1.099 to 1.320 in the primary model, whereas the corresponding ranges were 0.476–0.890 and 1.123–2.101 in the adjusted model. The maximum Cook’s distances were 0.033 and 0.080, respectively, indicating no influential observations.

**Figure 1:**
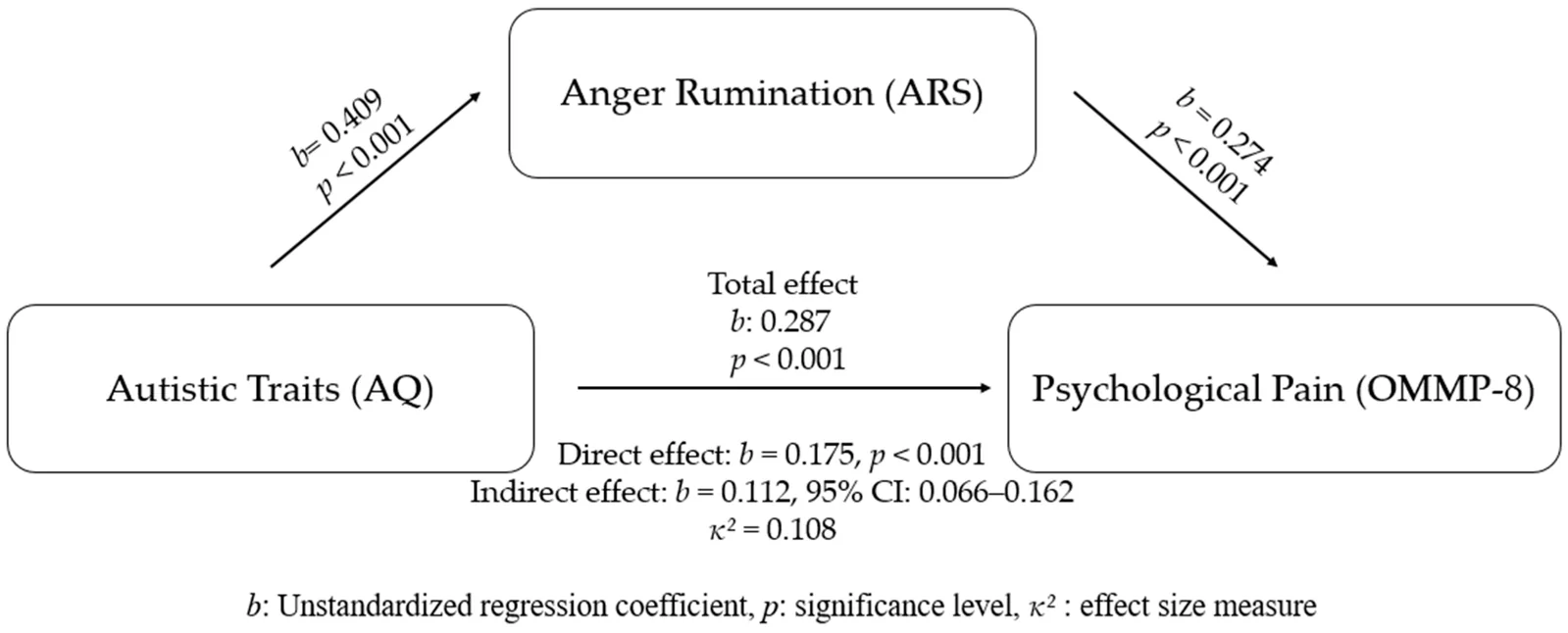
Indirect effect of autistic traits on psychological pain through anger rumination.

The model examining the indirect effect of autistic traits on psychological pain through anger rumination (ARS total) is shown in Figure 1. Autistic traits were significantly and positively associated with anger rumination (*b* = 0.409, *p* < 0.001), and anger rumination was significantly and positively associated with psychological pain (*b* = 0.274, *p* < 0.001). The total effect of autistic traits on psychological pain was significant (*b* = 0.287, *p* < 0.001), and the direct effect remained significant after accounting for anger rumination (*b* = 0.175, *p* < 0.001). The indirect effect through anger rumination was statistically significant (*b* = 0.112, 95% CI: 0.066–0.162, *κ*^2^ = 0.108), corresponding to approximately 39% of the total effect (Figure 1).

**Figure 2:**
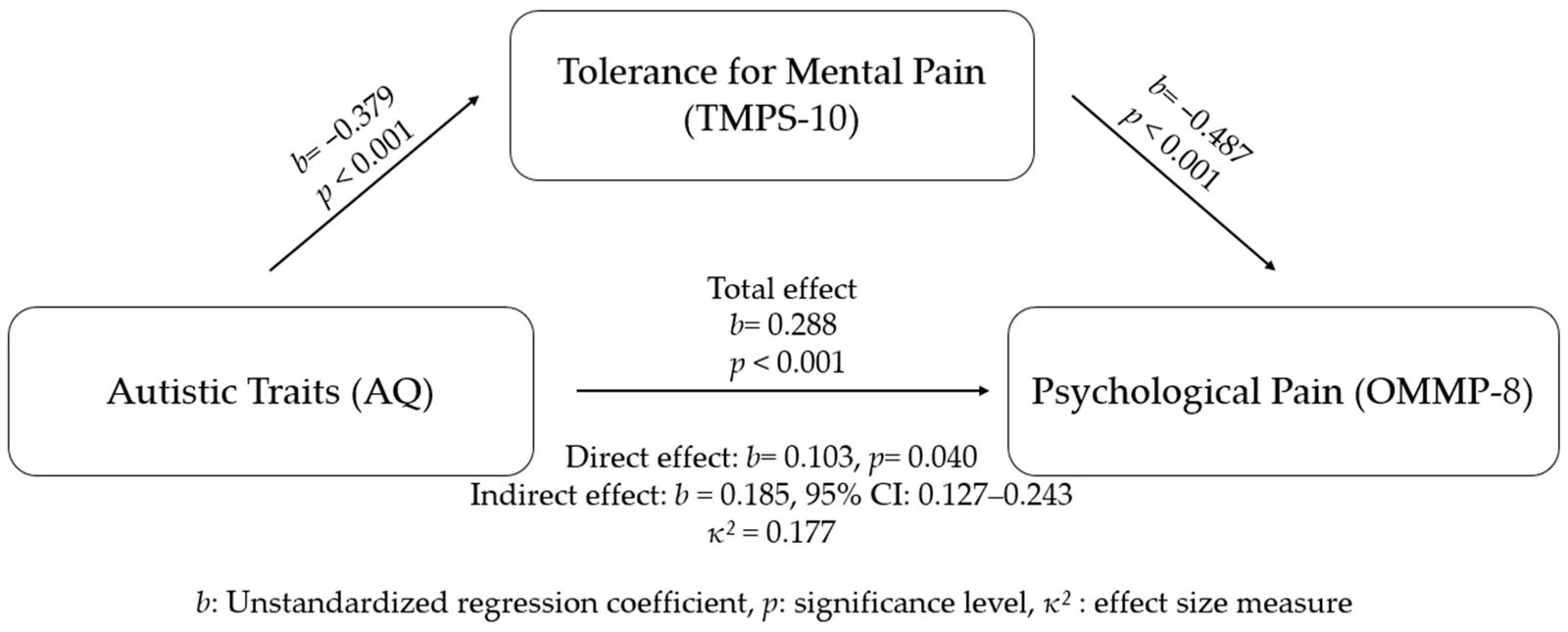
Indirect effect of autistic traits on psychological pain through tolerance for mental pain.

The model examining the indirect effect of autistic traits on psychological pain through tolerance for mental pain (TMPS-10 total) is shown in Figure 2. Autistic traits were significantly and negatively associated with tolerance for mental pain (*b* = −0.379, *p* < 0.001), and tolerance for mental pain was significantly and negatively associated with psychological pain (*b* = −0.487, *p* < 0.001). The total effect of autistic traits on psychological pain was significant (*b* = 0.288, *p* < 0.001), and the direct effect remained significant after accounting for tolerance for mental pain (*b* = 0.103, *p* = 0.040). The indirect effect through tolerance for mental pain was statistically significant (*b* = 0.185, 95% CI: 0.127–0.243, *κ*^2^ = 0.177), corresponding to approximately 64% of the total effect (Figure 2).

## 4. Discussion

This study examined the relationships among autistic traits, psychological pain, anger rumination, and tolerance for mental pain in medical students. The findings showed a significant positive relationship between autistic traits and psychological pain. In the regression analyses, autistic traits were significantly associated with psychological pain, while the mediation analyses showed that anger rumination and tolerance for mental pain had significant indirect effects on this relationship. However, in both models, the direct relationship between autistic traits and psychological pain remained significant after accounting for the respective mediator.

In the present study, participants with higher levels of autistic traits were found to have higher levels of psychological pain and lower tolerance for psychological pain. Significant associations were observed between the level of autistic traits and psychological pain, and regression analyses showed that autistic traits were significantly associated with psychological pain in both univariable and multivariable models. These findings support the relationship between autistic traits and psychological pain.

Previous studies have reported that autistic traits are commonly associated with depression, loneliness, social exclusion, and suicide risk (Dell’Osso et al., 2019; Cremone et al., 2024). Social isolation, exposure to bullying, and camouflaging autistic traits for the purpose of social adaptation may have adverse effects on psychological well-being (Carpita et al., 2025; Leedham et al., 2020; Cassidy et al., 2020). Similarly, reduced interpersonal communication and social isolation have been reported to be associated with psychological pain (Orbach et al., 2003).

In this context, interpersonal difficulties, social isolation, and perseverative thinking tendencies associated with autistic traits may be related to a more intense experience of psychological pain. Considering that medical students may constitute a psychologically vulnerable group due to intense academic workload, high performance expectations, and emotional stressors (Chandavarkar et al., 2007), these factors may be particularly relevant when considering the relationship between autistic traits and psychological pain in this population.

In the present study, anger rumination levels were found to increase as the level of autistic traits increased. In addition, participants with higher levels of autistic traits had higher levels of anger rumination. Interest in negative ruminative processes in autism has increased in recent years. However, studies examining anger rumination, which is defined as a perseverative cognitive process (Sukhodolsky et al., 2001), in the context of autistic traits remain limited (Patel et al., 2017).

Studies conducted with both individuals diagnosed with ASD and university students have reported that higher levels of autistic traits are associated with increased anger rumination (Patel et al., 2017; Pugliese et al., 2015). In addition, the relationship between autistic traits and anger rumination has been suggested to be linked to hostility and aggressive behaviors (Pugliese et al., 2015). It has also been proposed that the tendency toward perseveration associated with autistic traits may not be limited to behavioral repetitions, but may also include repetitive thought processes related to negative emotional experiences (Patel et al., 2017). Consistent with the literature, the findings of the present study support the notion that autistic traits may be associated with anger-related ruminative processes.

In the mediation analyses, anger rumination was found to have a significant indirect effect on the relationship between autistic traits and psychological pain. Moreover, the direct relationship between autistic traits and psychological pain remained significant after anger rumination was included in the model. In addition, regression analyses showed that anger rumination was significantly associated with psychological pain in both univariable and multivariable models.

Although anger rumination has previously been mostly associated with externalizing behaviors, such as physical and verbal aggression (Anestis et al., 2009; Peled & Moretti, 2010), an increasing number of studies in recent years have examined its relationship with internalizing processes, such as suicidal ideation, self-injurious behaviors, and psychological pain, in both clinical and non-clinical samples (Buerke & Capron, 2025; Wahba & Hamza, 2022; Kılıçaslan et al., 2022; Martino et al., 2015). In addition, a limited number of studies have reported that anger rumination levels increase as psychological pain increases (Uğur & Polat, 2021; Wahba & Hamza, 2022). Anger rumination appears to co-occur with repetitive thinking about negative emotional experiences, and these processes have been suggested to be associated with negative affect (Bushman et al., 2005).

Our findings are consistent with the previous literature reporting associations between autistic perseverative thinking tendencies and anger rumination (Patel et al., 2017), as well as between anger rumination and psychological pain (Uğur & Polat, 2021). The present findings extend this literature by showing a significant indirect effect of anger rumination in the association between autistic traits and psychological pain. Anger-related repetitive thinking may therefore be relevant to the association between higher levels of autistic traits and psychological pain.

Previous studies have reported that difficulties in cognitive flexibility and increased attention to detail, which may accompany autistic traits, can make it more difficult for individuals to disengage from negative thoughts and emotional experiences (Burrows et al., 2017). In this context, cognitive rigidity and difficulties in emotion regulation may be considered among the factors associated with reduced tolerance for psychological pain. Reduced tolerance for psychological pain, in turn, may be related to a more intense experience of psychological pain (Becker et al., 2019).

In the present study, a significant indirect effect of tolerance for psychological pain was observed in the association between autistic traits and psychological pain. This suggests that the association between autistic traits and psychological pain may also be considered in relation to individual differences in the capacity to tolerate psychological pain. Importantly, whereas the inverse relationship between tolerance for psychological pain and psychological pain has been previously reported (Becker et al., 2019), its relationship with autistic traits has not, to our knowledge, been directly examined. The negative association between tolerance for psychological pain and psychological pain observed in the multivariable analysis was consistent with this previously reported pattern.

Taken together, the indirect effects corresponded to approximately 39% and 64% of the total effects in the anger rumination and tolerance for psychological pain models, respectively, indicating a relatively larger indirect effect for tolerance for psychological pain. Although both indirect effects were statistically significant, these findings do not establish temporal ordering among the variables or provide evidence of causal mediation mechanisms.

In our sample of medical students, the observed associations may contribute to a better understanding of psychological pain and related cognitive and emotional characteristics in students with higher levels of autistic traits. In particular, the findings suggest that anger rumination and tolerance for psychological pain may be considered cognitive and emotional characteristics associated with psychological pain in this population. Further longitudinal and intervention studies are needed to determine whether these findings can inform preventive or supportive approaches for medical students.

One of the important strengths of the present study is that it examined the relationships among autistic traits, psychological pain, anger rumination, and tolerance for mental pain together. To the best of our knowledge, the number of studies evaluating these variables together, particularly within mediation models, is highly limited. In addition, the use of a relatively large sample and the inclusion of both preclinical and clinical-year medical students are important in terms of capturing students at different stages of medical education.

However, this study has several limitations. First, the cross-sectional design of the study does not allow causal relationships between variables to be evaluated. The fact that the study was conducted at a single center and included only medical students may limit the generalizability of the findings. In addition, all main variables were assessed using self-report measures administered at the same time, which may have increased the risk of response bias and common method variance. Moreover, given the conceptual proximity between psychological pain and tolerance for psychological pain, as assessed by the OMMP-8 and TMPS-10, respectively, the possibility of some degree of construct overlap cannot be excluded. Accordingly, associations involving these measures should be interpreted with this potential overlap in mind. In the present study, the Cronbach’s alpha coefficients obtained for the AQ total score and the “Enduring the Pain” subdomain of the TMPS-10 were relatively low. However, the TMPS-10 total score was used in the main regression and mediation analyses, and the subdomain with low reliability was not evaluated as an independent variable. Furthermore, autistic traits were assessed using a self-report screening scale, and no clinical diagnostic evaluation was performed. The mediation analyses were conducted using cross-sectional data and therefore do not demonstrate causality. Finally, although psychological pain is known to be associated with suicidal processes, current suicidal ideation and suicidal behaviors were not directly assessed in the present study, which should be considered an important limitation.

## 5. Conclusions

The findings of the present study showed that autistic traits were associated with higher levels of psychological pain and anger rumination and lower tolerance for mental pain among medical students. In addition, both anger rumination and tolerance for mental pain had significant indirect effects on the relationship between autistic traits and psychological pain. These findings highlight the relevance of anger rumination and tolerance for mental pain to the relationship between autistic traits and psychological pain.

To the best of our knowledge, this study is among the limited number of studies that directly examine the relationship between autistic traits and psychological pain, and it provides an original contribution by evaluating this relationship in the context of anger rumination and tolerance for mental pain. These findings highlight the importance of assessing psychological pain, anger rumination, and tolerance for mental pain in individuals with higher levels of autistic traits.

Future longitudinal studies and studies conducted in clinical samples may contribute to a better understanding of these relationships, as well as their associations with suicidal ideation and suicidal behavior.

## Figures and Tables

**Table 1:** Sociodemographic and Clinical Characteristics of the Participants.

Sociodemographic Characteristics			Clinical Characteristics		
	**Mean ± SD**				***n* (%)**
Age	22.40 ± 1.91		Psychiatric history (Yes)	Current psychiatric disorder	42(7.7)
		***n* (%)**		History of psychiatric disorder	63(11.6)
Classification Group	Preclinical	291(53.5)		History of suicide attempt	12(2.2)
	Clinical	253(46.5)		Family history of psychiatric disorder	66(12.1)
Sex	Male	243(44.7)		Psychotropic medication use	31(5.7)
	Female	301(55.3)			
Marital Status	Single	537(98.7)	Number of suicide attempts	1	9(1.7)
	Married	7(1.3)		≥2	3(0.6)
Place of Residence	Student house	325(59.7)	Method of suicide attempt	Medication overdose	4(0.7)
	State dormitory	137(25.2)		Sharp object injury	6(1.1)
	Family home	61(11.2)		Other (jumping from a height, jumping in front of a vehicle, etc.)	2(0.4)
	Private dormitory	21(3.9)			

Frequencies and descriptive statistics are presented as *n* (%) or mean ± SD. Mean: arithmetic mean; SD: standard deviation. Percentages were calculated based on *N* = 544.

**Table 2:** Descriptive Statistics and Comparisons of Scale Scores According to Autism Spectrum Quotient Groups.

		TOTAL *N* = 544		AQ < 26 *n* = 446	AQ ≥ 26 *n* = 98	
		Mean ± SD	Min–Max	Mean ± SD	Mean ± SD	*p*
**AQ**		20.61 ± 5.38	7–38	18.89 ± 4.15	28.44 ± 2.79	<0.001
**OMMP-8**	Total	10.86 ± 5.60	0–27	10.48 ± 5.42	12.59 ± 6.11	0.001
	Factor 1	1.56 ± 1.78	0–8	1.38 ± 1.71	2.35 ± 1.88	<0.001
	Factor 2	6.87 ± 2.94	0–12	6.84 ± 2.92	7.00 ± 3.05	0.619
	Factor 3	2.44 ± 2.51	0–12	2.26 ± 2.46	3.24 ± 2.62	<0.001
**TMPS-10**	Total	36.26 ± 7.05	11–50	37.01 ± 6.86	32.82 ± 6.87	<0.001
	EP	18.92 ± 4.37	5–25	19.33 ± 4.26	17.01 ± 4.40	<0.001
	MP	17.34 ± 4.21	5–25	17.68 ± 4.14	15.81 ± 4.19	<0.001
**ARS**	Total	44.04 ± 10.93	18–72	43.56 ± 10.68	46.26 ± 11.84	0.027
	Understanding the Reasons	10.12 ± 2.73	4–16	10.00 ± 2.68	10.70 ± 2.91	0.020
	Angry Memories	12.92 ± 3.54	5–20	12.78 ± 3.46	13.59 ± 3.81	0.039
	Thoughts of Revenge	7.83 ± 2.61	4–16	7.71 ± 2.53	8.40 ± 2.88	0.017
	Angry Afterthoughts	15.69 ± 4.43	6–24	15.58 ± 4.40	16.18 ± 4.55	0.219

Mean ± SD: mean ± standard deviation; Min–Max: minimum–maximum; AQ: Autism Spectrum Quotient; OMMP-8: Orbach and Mikulincer Mental Pain Scale-8; TMPS-10: Tolerance for Mental Pain Scale-10; EP: Enduring the Pain; MP: Managing the Pain; ARS: Anger Rumination Scale. An independent-samples t-test was used for group comparisons. Statistical significance was set at *p* < 0.05.

**Table 3:** Pearson Correlations Between AQ Total Scores and OMMP-8, TMPS-10, and ARS Scores.

		AQ Total	
		(*N* = 544)	
		*r*	*p*
**OMMP-8**	Total	0.276 **	<0.001
	Factor 1	0.294 **	<0.001
	Factor 2	0.099 *	0.020
	Factor 3	0.291 **	<0.001
**TMPS-10**	Total	−0.289 **	<0.001
	EP	−0.251 **	<0.001
	MP	−0.223 **	<0.001
**ARS**	Total	0.202 **	<0.001
	Understanding the Reasons	0.194 **	<0.001
	Angry Memories	0.182 **	<0.001
	Thoughts of Revenge	0.172 **	<0.001
	Angry Afterthoughts	0.163 **	<0.001

Pearson correlation coefficients (*r*) are presented. * *p* < 0.05, ** *p* < 0.01. AQ: Autism Spectrum Quotient; OMMP-8: Orbach and Mikulincer Mental Pain Scale-8; TMPS-10: Tolerance for Mental Pain Scale-10; EP: Enduring the Pain; MP: Managing the Pain; ARS: Anger Rumination Scale.

**Table 4:** Simple and Multiple Linear Regression Analyses of Variables Associated with OMMP-8 Total Scores.

**Independent Variables**	**Unstandardized Coefficients**		**Standardized Coefficients**	***t***	***p***	**95% Confidence Interval**			
	***B***	**SE**	***β***			**Lower**	**Upper**	***R* ^2^**	
**ARS-Total**	0.291	0.018	0.568	15.904	<0.001	0.255	0.327	0.323	
**TMPS-10-Total**	−0.510	0.027	−0.641	−18.933	<0.001	−0.563	−0.457	0.412	
**AQ-Total**	0.287	0.041	0.276	7.011	<0.001	0.207	0.367	0.076	
**Multiple Linear Regression Analysis**									
**Independent Variables**	**Unstandardized Coefficients**		**Standardized Coefficients**	***t***	***p***	**95% Confidence Interval**		**Tolerance**	**VIF**
	***B***	**SE**	***β***			**Lower**	**Upper**		
**Constant**	14.977	1.700		8.808	<0.001	11.637	18.318		
ARS-Total	0.177	0.017	0.345	10.233	<0.001	0.143	0.210	0.793	1.261
TMPS-10-Total	−0.370	0.027	−0.466	−13.528	<0.001	−0.424	−0.317	0.757	1.320
AQ-Total	0.075	0.033	0.072	2.288	0.023	0.011	0.139	0.910	1.099
Model 1: *R*^2^ = 0.515; adjusted *R*^2^ = 0.512; *F* = 190.832; *p* < 0.001; Durbin–Watson = 1.867.									
**Adjusted Multiple Linear Regression Analysis**									
**Independent Variables**	**Unstandardized Coefficients**		**Standardized Coefficients**	***t***	***p***	**95% Confidence Interval**		**Tolerance**	**VIF**
	***B***	**SE**	***β***			**Lower**	**Upper**		
**Constant**	14.736	1.705	-	8.643	<0.001	11.387	18.085	-	-
ARS-Total	0.175	0.017	0.342	10.065	<0.001	0.141	0.209	0.779	1.284
TMPS-10-Total	−0.365	0.028	−0.460	−13.188	<0.001	−0.420	−0.311	0.740	1.352
AQ-Total	0.078	0.033	0.075	2.370	0.018	0.013	0.143	0.890	1.123
Current psychiatric disorder	−0.747	0.911	−0.036	−0.820	0.413	−2.536	1.043	0.476	2.101
History of psychiatric disorder	−0.019	0.558	−0.001	−0.034	0.973	−1.116	1.078	0.881	1.135
Psychotropic medication use	2.058	1.042	0.085	1.975	0.049	0.011	4.106	0.482	2.075
History of suicide attempt	−0.559	1.227	−0.015	−0.455	0.649	−2.969	1.851	0.866	1.154
Adjusted model: *R*^2^ = 0.518; adjusted *R*^2^ = 0.512; *F* = 82.446; *p* < 0.001; Durbin–Watson = 1.874.									

Dependent variable: OMMP-8 total score. SE: standard error; VIF: variance inflation factor; AQ: Autism Spectrum Quotient; OMMP-8: Orbach and Mikulincer Mental Pain Scale-8; TMPS-10: Tolerance for Mental Pain Scale; ARS: Anger Rumination Scale.

## Data Availability

The data that support the findings of this study are available from the corresponding author upon reasonable request.

## Supplementary Materials

The following supporting information can be downloaded at: https://www.mdpi.com/article/10.3390/bs16101824/s1.
