# Supplementary material for: Psychological Pain and Its Associations with Autistic Traits, Anger Rumination, and Tolerance for Mental Pain Among Medical Students
Source: Behav Sci (Basel). 2026 Oct 6;16(10):1824. doi: 10.3390/bs16101824 (PMC13701755; doi:10.3390/bs16101824)
Supplement: Supplementary file 1 [file behavsci-16-01824-s001.zip › behavsci-4574542-supplementary.pdf]

STROBE Statement—Checklist of items that should be included in reports of observational studies

	Item No	Recommendation	Reported on page no.
Title and abstract	1	(a) Indicate the study's design with a commonly used term in the title or the abstract	1
Title and abstract	1	(b) Provide in the abstract an informative and balanced summary of what was done and what was found	1
**Introduction**	**Introduction**	**Introduction**	
Background/rationale	2	Explain the scientific background and rationale for the investigation being reported	2–3
Objectives	3	State specific objectives, including any prespecified hypotheses	3
**Methods**	**Methods**	**Methods**	
Study design	4	Present key elements of study design early in the paper	4
Setting	5	Describe the setting, locations, and relevant dates, including periods of recruitment, exposure, follow-up, and data collection	4
Participants	6	(a) Cohort study—Give the eligibility criteria, and the sources and methods of selection of participants. Describe methods of follow-up<br>Case-control study—Give the eligibility criteria, and the sources and methods of case ascertainment and control selection. Give the rationale for the choice of cases and controls<br>Cross-sectional study—Give the eligibility criteria, and the sources and methods of selection of participants	4
Participants	6	(b) Cohort study—For matched studies, give matching criteria and number of exposed and unexposed<br>Case-control study—For matched studies, give matching criteria and the number of controls per case	N/A
Variables	7	Clearly define all outcomes, exposures, predictors, potential confounders, and effect modifiers. Give diagnostic criteria, if applicable	4–6
Data sources/ measurement	8*	For each variable of interest, give sources of data and details of methods of assessment (measurement). Describe comparability of assessment methods if there is more than one group	4–6
Bias	9	Describe any efforts to address potential sources of bias	4
Study size	10	Explain how the study size was arrived at	4
Quantitative variables	11	Explain how quantitative variables were handled in the analyses. If applicable, describe which groupings were chosen and why	5-6
Statistical methods	12	(a) Describe all statistical methods, including those used to control for confounding	6-7
Statistical methods	12	(b) Describe any methods used to examine subgroups and interactions	6
Statistical methods	12	(c) Explain how missing data were addressed	N/A
Statistical methods	12	(d) Cohort study—If applicable, explain how loss to follow-up was addressed<br>Case-control study—If applicable, explain how matching of cases and controls was addressed<br>Cross-sectional study—If applicable, describe analytical methods taking account of sampling strategy	N/A
Statistical methods	12	(e) Describe any sensitivity analyses	N/A

**Results**	**Results**	**Results**	**Reported on page no.**
Participants	13*	(a) Report numbers of individuals at each stage of study—eg numbers potentially eligible, examined for eligibility, confirmed eligible, included in the study, completing follow-up, and analysed	4
Participants	13*	(b) Give reasons for non-participation at each stage	4
Participants	13*	(c) Consider use of a flow diagram	N/A
Descriptive data	14*	(a) Give characteristics of study participants (eg demographic, clinical, social) and information on exposures and potential confounders	7-8
Descriptive data	14*	(b) Indicate number of participants with missing data for each variable of interest	N/A
Descriptive data	14*	(c) Cohort study—Summarise follow-up time (eg, average and total amount)	N/A
Outcome data	15*	Cohort study—Report numbers of outcome events or summary measures over time	
Outcome data	15*	Case-control study—Report numbers in each exposure category, or summary measures of exposure	
Outcome data	15*	Cross-sectional study—Report numbers of outcome events or summary measures	8–9
Main results	16	(a) Give unadjusted estimates and, if applicable, confounder-adjusted estimates and their precision (eg, 95% confidence interval). Make clear which confounders were adjusted for and why they were included	4,10-12
Main results	16	(b) Report category boundaries when continuous variables were categorized	5,8-9
Main results	16	(c) If relevant, consider translating estimates of relative risk into absolute risk for a meaningful time period	N/A
Other analyses	17	Report other analyses done—eg analyses of subgroups and interactions, and sensitivity analyses	8-9
**Discussion**	**Discussion**	**Discussion**	
Key results	18	Summarise key results with reference to study objectives	14
Limitations	19	Discuss limitations of the study, taking into account sources of potential bias or imprecision. Discuss both direction and magnitude of any potential bias	17
Interpretation	20	Give a cautious overall interpretation of results considering objectives, limitations, multiplicity of analyses, results from similar studies, and other relevant evidence	14-17

Generalisability	21	Discuss the generalisability (external validity) of the study results	17
**Other information**	**Other information**	**Other information**	
Funding	22	Give the source of funding and the role of the funders for the present study and, if applicable, for the original study on which the present article is based	18

N/A: not applicable to the present cross-sectional study or analysis.
